# Disease Course, Management and Outcomes in Kidney Transplant Recipients with SARS-CoV-2 Infection during the Omicron-Variant Wave: A Single-Center Experience

**DOI:** 10.3390/vaccines11030632

**Published:** 2023-03-12

**Authors:** Maria Korogiannou, Kalliopi Vallianou, Efstathios Xagas, Evangelia Rokka, Ioanna Soukouli, Ioannis N. Boletis, Smaragdi Marinaki

**Affiliations:** Clinic of Nephrology and Renal Transplantation, Laiko General Hospital, Medical School of Athens, National and Kapodistrian University, 11527 Athens, Greece; kvallia@med.uoa.gr (K.V.); exagas@med.uoa.gr (E.X.); liliarokka@med.uoa.gr (E.R.); laikneph@laiko.gr (I.S.); inboletis@med.uoa.gr (I.N.B.); smarinak@med.uoa.gr (S.M.)

**Keywords:** COVID-19, kidney transplantation, Omicron wave, SARS-CoV-2, vaccination

## Abstract

Background: Since December 2019, kidney transplant recipients (KTRs) have experienced a great impact of the coronavirus disease 2019 (COVID-19) pandemic, with a higher risk of morbidity and mortality compared to the general population. Preliminary data in KTRs suggest that the Omicron variant, which has been dominant since December 2021, is more infectious than the previous ones but is associated with reduced risk of severity and low lethality rates. The purpose of our study was to assess the disease course and outcomes of the SARS-CoV-2 infection in KTRs during the Omicron-surge. Methods: This retrospective study included 451 KTRs diagnosed with SARS-CoV-2 infection between 1 December 2021 and 30 September 2022. Demographic and clinical characteristics at the time of infection, vaccination data, treatment, clinical course, and outcomes were recorded and analyzed. Results: Mean age was 51.8 ± 13.7 years with a male predominance (61.2%). The majority (76.1%) were vaccinated with at least three doses of the available mRNA vaccines, although serology revealed low anti-SARS-CoV-2 antibody titers before infection (33 [3.3–1205] AU/mL). Only 6% of the patients experienced moderate–severe disease. Accordingly, there was low prevalence of adverse outcomes, such as SARS-CoV-2-related hospitalization (11.3%) and death (0.9%). Multivariate analysis revealed that only age significantly increased the risk of SARS-CoV-2-related hospitalization. Conclusions: During the Omicron wave, the clinical course of the SARS-CoV-2 infection in KTRs has substantially changed, with lower rates of moderate and severe disease and a low prevalence of adverse outcomes. Prospective clinical trials are warranted to further elucidate the evolving pathogenesis, management, and long-term outcomes of COVID-19 in such high-risk populations.

## 1. Introduction

Since early 2020, severe acute respiratory syndrome coronavirus 2 (SARS-CoV-2) has spread rapidly around the world, causing the loss of unprecedented numbers of lives. Kidney transplant recipients (KTRs) have experienced a great impact of the coronavirus disease 2019 (COVID-19) pandemic, with a higher risk of morbidity and mortality compared to the general population [1,2]. The burden of immunosuppression and presence of comorbidities in the population of solid organ transplant (SOT) recipients have been related with an increased risk of complications from COVID-19 [3]. 

In the first wave of the pandemic, before vaccines and specific anti-SARS-CoV-2 treatments were available, the overall mortality reported in SOT recipients ranged 19–50% [3,4,5], which is up to 10-fold higher compared to the general population.

Recently, the Omicron variant which has been dominant since December 2021, has shown increased transmission rates and less severity in the general population after two vaccinations, compared with the Delta variant [6]. The same appears to be the case for SOT, with studies showing increased infection rates and low hospitalization and mortality rates compared with data from earlier in the pandemic [7,8]. Particularly in KTRs, the risk of breakthrough infection with the Omicron variant appears to further increase, considering the reduced humoral response observed in KTRs after vaccination [9,10,11] and higher risk of breakthrough infection in vaccinated SOT recipients compared to the general population [12,13]. Indeed, preliminary data in kidney transplant recipients suggest that the Omicron variant is more infectious than the previous reported variants but is associated with a reduced risk of severity and low lethality rates [7]. Nevertheless, severity and mortality of the disease still remain higher than in the general population [8].

Therefore, the purpose of our study was to further elucidate the epidemiology, clinical course, therapeutic management, and outcomes of COVID-19 infection in KTRs during the third wave of the pandemic, when the Omicron variant was predominant in Greece.

## 2. Materials and Methods

This observational retrospective study was conducted at the Clinic of Nephrology and Renal Transplantation, Laiko General Hospital of Athens, Greece, aiming to assess the disease course and clinical outcomes of SARS-CoV-2 infection during the Omicron surge in KTRs.

All adult (≥18 years old) KTRs actively followed at our clinic who were diagnosed with SARS-CoV-2 infection by real time polymerase chain reaction (PCR) between 1 December 2021 and 30 September 2022 were included in the analysis. The SARS-CoV-2 genotyping assays were not available, so our study included all patients with a positive PCR-test for SARS-CoV-2 during the defined period of time, when according to the epidemiology of the disease in Greece the Omicron variant was predominant. The PCR-testing for SARS-CoV-2 was performed when any symptom of the disease appeared, such as fever, cough, malaise, myalgias, or even less common symptoms such as diarrhea, but also in asymptomatic patients, considering that in Greece PCR-testing was routinely performed for several activities during that period of time. 

Clinical data on transplantation status, immunosuppression, chronic comorbidities, and concomitant medications, as well as adjustment of immunosuppressive regimen during SARS-CoV-2 infection, vaccination status against SARS-CoV-2 and humoral response after vaccination, specific SARS-CoV-2 treatments, and complications related to COVID-19, were obtained from medical charts. All laboratory tests including full blood count, ferritin, C-reacting protein, serum creatinine and levels of immunosuppressive medications were performed in the central laboratory of our hospital upon diagnosis of SARS-CoV-2 infection and were subsequently recorded in the patients’ charts. Estimated glomerular filtration rate (eGFR) was calculated using the Chronic Kidney Disease Epidemiology Collaboration (CKD-EPI) equation. Acute kidney injury (AKI) was defined according to the 2012 Kidney Disease Improving Global Outcome (KDIGO) guidelines.

Vaccinated KTRs had received either of the two authorized mRNA SARS-CoV-2 vaccines (BNT162b2, Pfizer-BioNTech or mRNA1273, Moderna). Antibody titers before infection, when available, were defined as those measured at a median of 28 days after the last vaccine dose and before the infection occurred. They were measured utilizing a chemiluminescent microparticle immune assay (CMIA) which quantifies IgG antibodies against the receptor-binding domain (RBD) of the S1 subunit of the spike protein of SARS-CoV-2 (Abbott SARS-CoV-2 IgG II Quant). The linear range of the assay is between 21 and 40,000 Arbitrary Units per milliliter (AU/mL) and the lower limit of detection (LoD) 6.8 AU/mL. Anti-SARS-CoV-2 receptor-binding domain IgG assays have shown an excellent correlation with neutralizing antibodies [14].

The study was approved by the Ethics committee of Laiko General Hospital (Protocol Number 212/20-01-23).

### 2.1. Definition of COVID-19 Severity

According to the FDA, mild disease is characterized by fever, malaise, cough, myalgias, anosmia and ageusia and other less common symptoms (i.e., nausea, vomiting or diarrhea in the absence of low respiratory tract involvement). Moderate disease includes lower respiratory tract involvement with infiltrates on chest imaging, without dyspnea or hypoxia. Finally severe disease is defined as presence of lung infiltrates with hypoxia-oxygen saturation at room air less than 94% and/or need for oxygen supplementation or ventilator support.

### 2.2. Statistical Analysis

Statistical analysis was performed with STATA v13.1 software (StataCorp LLC, College Station, TX, USA). The normality of distribution for quantitative variables was examined using the Shapiro–Wilk test. Continuous variables are presented as mean ± standard deviation (mean ± SD) or median (interquartile range), according to the normality of distribution. Categorical variables are presented as absolute frequencies and percentages (*n*, %). Finally, multivariate logistic regression analysis was performed to identify factors associated with need for hospitalization. Odds ratios (OR) and 95% confidence internals (CI) were calculated to evaluate the strength of any association. A *p*-value of < 0.05 (two-tailed) was considered statistically significant.

## 3. Results

### 3.1. Baseline Characteristics

Table 1 presents baseline demographics, clinical and laboratory characteristics, as well as main comorbidities and immunosuppressive treatment of the study population.

Overall, 451 consecutive KTRs who were diagnosed with SARS-CoV-2 infection between 1 December 2021 and 30 September 2022 during the Omicron surge, were included in the analysis. Mean age of the study population was 51.8 ± 13.7 years and there was a male predominance (61.2%). Median BMI was 25 (22.5–27.5) kg/m^2^ and median time from transplantation was 6.6 (2.7–15) years and median baseline serum creatinine was 1.4 (1.14–1.76) mg/dL (median e GFR: 50 (40–69) mL/min/1.73 m^2^). Regarding major comorbidities, the majority of patients suffered from hypertension (72.9%), whereas diabetes mellitus and cardiovascular disease were less common (12.1% and 14.8%, respectively). Most patients (72.3%) were on a triple calcineurin inhibitor (CNI)-based immunosuppressive regimen, mainly tacrolimus (81.6%), in combination with mycophenolate mofetil/mycophenolic acid (MMF/MPA) (90.7%) and corticosteroids (87.1%).

### 3.2. Vaccination Status and SARS-CoV-2 Serology

Vaccination status and anti-SARS-CoV-2 RBD-IgG antibody titers before infection are presented in Table 2. A total of 390 KTRs (86.4%) were vaccinated with two doses of the vaccine, most of whom had also received a third dose (76.1% of total) and 32.8% had received a fourth dose before infection. Time from vaccination with two doses of the available vaccines at the time of infection was 294 (257–382) days, with three doses was 188 (125–266) days and with four doses was 27 (13–33) days. Among 108 patients for whom serology was available, the median anti-SARS-COV-2 RBD-IgG antibody titers were 33 (3.3–1205) AU/mL before infection. 

### 3.3. Disease Course and Outcomes

Disease course, clinical and laboratory findings during infection with SARS-CoV-2 and outcomes are depicted in Table 3. Overall, 22 out of 451 patients were re-infected with SARS-CoV-2. Twenty-six patients (6%) experienced moderate and severe disease, according to presence of pathologic radiographic findings. In total, 51 (11.3%) of the study population were hospitalized, of whom 52.9% (27 patients) were hospitalized for COVID-19 related symptoms (i.e., fever, pneumonia, diarrhea). The need for oxygen supplementation was reported in 21 patients (4.6%); only 1.7% of patients needed mechanical ventilation and were admitted in the Intensive Care Unit (ICU) and death from SARS-CoV-2 was reported in 4 patients (0.9%).

Regarding laboratory findings, the median value of white blood cells (WBC) was 7255 (5420–9400) cells/10^3^ and the median number of lymphocytes was 1269 (740–1880) cells/10^3^. The levels of acute phase proteins ferritin and C-reacting protein (CRP) were 236 (120–550) ng/mL and 4 (1.4–14.5) mg/L, respectively. Renal function was well preserved, with only 4% of patients experiencing AKI.

### 3.4. Baseline Characteristics and Outcomes of Patients with Moderate and Severe Disease

A sub-group analysis of the 26 patients of the study population who experienced moderate or severe disease is presented in Table 4 and Table 5. 

The mean age of patients with moderate and severe disease was 63.5 ± 9.2 years and there was a male predominance (61.5%). The median BMI was 26.1 (21.5–27.9) kg/m^2^ and median time from transplantation was 8.1 (3.7–13.7) years and median baseline serum creatinine was 1.3 (1.2–1.8) mg/dL (median e GFR: 46 (37–59) mL/min/1.73 m^2^). Regarding major comorbidities, the majority of patients suffered from hypertension (84.6%), whereas diabetes mellitus and cardiovascular disease were less common (26.9% and 19.2%, respectively). Most patients were on a triple calcineurin inhibitor (CNI)-based immunosuppressive regimen, mainly tacrolimus (80.8%), in combination with mycophenolate mofetil/mycophenolic acid (MMF/MPA) (84.6%) and corticosteroids (92.3%).

Regarding laboratory findings, the median value of white blood cells (WBC) was 7520 (4700–10,350) cells/10^3^ and the median number of lymphocytes was 640 (440–1020) cells/10^3^. The levels of acute phase proteins ferritin and C-reacting protein (CRP) were 652 (209–1063) ng/mL and 42 (16–69.1) mg/L, respectively. Renal function was well preserved, with only 11.5% of patients experiencing AKI. In total, 21 (80.8%) of the study population were hospitalized, all of whom for COVID-19 related symptoms (i.e., fever, pneumonia, diarrhea). The need for oxygen supplementation was reported in 18 patients (69.2%), while five patients (19.2%) of patients needed mechanical ventilation and were admitted in the Intensive Care Unit (ICU) and death was reported in two patients (7.7%).

Apart from the above-mentioned results, our analysis also showed that patients who experienced moderate or severe disease had more severe lymphopenia (*p* < 0.05) and higher levels of ferritin (*p* < 0.05) and CRP (*p* < 0.05) compared with patients with mild disease, as expected. Moreover, multivariate analysis of factors associated with disease severity, such as age, gender, years from transplantation, hypertension, diabetes, BMI, and vaccination status revealed that only age was significantly associated with disease severity (OR 1.11, *p* = 0.001). There was also an association of BMI with disease severity, although it did not reach statistical significance (OR 1.16, *p* = 0.060).

### 3.5. Therapeutic Management

Table 6 describes therapeutic management of the disease. A reduction in the intensity of immunosuppressive treatment was applied in all patients, according to previous strategies with other variants applied in our center, which were based on expert opinion papers [15,16]. In 338 out of 409 patients receiving MMF/MPA (82.6%), the antimetabolite was discontinued, and in 17.4% who were at an increased immunologic risk and experienced mild disease symptoms, there was a dose reduction. A reduction in tacrolimus with target trough levels below 6 ng/mL was reported in 150 out of 386 patients receiving tacrolimus. In those who recovered, immunosuppression was gradually restored to pre-infection levels after 3 weeks.

Treatment of SARS-CoV-2 infection consisted of Dexamethasone in 20 patients (4.4%) and a 5-day Remdesivir course in 18 patients (3.9%), all of whom were hospitalized and had hypoxia. Only two patients with severe disease received Tocilizumab (0.4%). Depending on availability of therapeutic agents, asymptomatic and oligosymptomatic patients received a 3-day Remdesivir course (40.6%), Molnupiravir (21.5%) or monoclonal antibodies (10.4%). The vast majority of patients were prescribed anticoagulants (86.6%) for 3 weeks, independently of disease severity and broad-spectrum antibiotics were administered in 33 (7.3%) patients. 

### 3.6. Factors Associated with Need for Hospitalization

Multivariate analysis of variables associated with the need for hospitalization during the Omicron wave revealed that for each year of age, the risk of hospitalization increased in our population by 6% and this increase was statistically significant (*p* = 0.001). Accordingly, for each year since the transplantation procedure, the risk decreased by 2%, though this association was not statistically significant.

Comorbidities (hypertension and diabetes mellitus) were associated with an increased risk for hospitalization, without reaching statistical significance, while BMI and renal function in our population were not found to be associated with the risk of hospitalization. Vaccination against SARS-CoV-2 was associated with a reduced risk for hospitalization (OR 0.52), but this finding was again not statistically significant (*p* = 0.471). Finally, treatment with either Remdesivir, Molnupiravir or monoclonal antibodies was not associated with a reduction in risk for hospitalization.

## 4. Discussion

This retrospective study aimed to describe epidemiologic data, clinical course, and outcomes of SARS-CoV-2 infection in a large cohort of KTRs during the third wave of the pandemic, when the Omicron variant was dominant and after new preventive and therapeutic measures had become available. 

The vast majority of our cohort (76.1%) were vaccinated with at least three doses of the two available mRNA vaccines, although serology, when available, revealed low anti-SARS-CoV-2 antibody titers before infection. Interestingly, we found that a low proportion of patients (6%) experienced moderate and severe disease. Accordingly, there was a low prevalence of adverse outcomes related to COVID-19, such as SARS-CoV-2-related hospitalization and death. Finally, multivariate analysis of variables associated with the need for hospitalization during the Omicron wave revealed that only age increased the risk of SARS-CoV-2-related hospitalization, whereas treatment with either Remdesivir, Molnupiravir or monoclonal antibodies was not associated with a reduction in risk for hospitalization.

Evolution of the SARS-CoV-2 pandemic and the high prevalence of the Omicron variant during the third wave worldwide, has emerged new challenges regarding the prevention and control of the infection. Our study revealed low rates of hospitalization and mortality (11.3% and 0.9%, respectively). These findings come in agreement with what has been described both in the general population [17] and in KTRs [7,18]. Recently published United States data in 347 SOT recipients during the Omicron surge, revealed a reduction in rates of hospitalization (26% vs. 60%) and mortality (2% vs. 10%), as compared with the first and second wave of the pandemic [7]. Similarly, results from a French single-kidney transplantation center in a cohort of 860 fully vaccinated KTRs during the Omicron outbreak showed a mortality rate as low as 4% [18]. The reduced rates of adverse outcomes could be explained by low pathogenicity of the Omicron variant along with the availability of more efficient preventive and therapeutic management.

Another important finding of our study is that the majority of the infected KTRs were vaccinated with at least three doses of the available mRNA vaccines and had low levels of anti-SARS-CoV-2 antibody titers before time of infection, despite the administration of booster doses (3rd and 4th dose). Though existing data indicate that protection against infection with the Omicron variant in KTRs can be increased by administering booster doses [19], there are other studies that have shown a rather poor neutralizing response to the Omicron variant one month after completion of mRNA vaccines [20,21]. This may explain why vaccinated KTRs remain at high risk of breakthrough infection with the Omicron variant. Nevertheless, protection against severe disease might still exist and be associated with vaccine-induced cellular immunity [22].

A reduction in the intensity of immunosuppressive treatment was applied to all patients, most commonly with discontinuation of the antimetabolite. Management of immunosuppression during the Omicron surge in our transplantation center followed previous recommendations by the DESCARTES Working Group [15]. Considering that both our findings and the results of several studies in KTRs during the third wave suggest a milder course of the disease, no adjustment of immunosuppressive therapy could be proposed for asymptomatic, ambulatory patients. 

Regarding therapeutic options, monoclonal antibodies have shown to be safe in KTRs, with no definitive data on their efficacy in the transplant population. This could be explained by the fact that KTRs were not included in randomized control trials with monoclonal antibodies and the only data available come from retrospective studies and case series [23,24,25]. In a retrospective study by Klein et al. in 95 KTRs, monoclonal antibodies were administered in 20 patients to whom there was a significant risk in hospitalizations and visits in the emergency department [26]. Another recent retrospective study on 243 KTRs aiming to describe the evolving epidemiology and outcomes of PCR-documented SARS-CoV-2 infection, the early administration of monoclonal antibodies was associated with a better outcome [27]. With regards to antiviral agents, studies on the general population have shown that they are effective in the treatment of non-hospitalized patients with COVID-19. The administration of a 3-day course of Remdesivir on non-hospitalized patients at high risk for COVID-19 progression has demonstrated an acceptable safety profile and resulted in a 87% lower risk of hospitalization or death compared with placebo in a randomized placebo-controlled trial that included 562 patients [28]. Moreover, in another randomized placebo-controlled trial on 1433 unvaccinated patients, initiation of Molnupiravir within 5 days after onset of mild to moderate disease reduced the risk of hospitalization or death [29]. In our analysis, treatment of SARS-CoV-2 infection, depending on availability of treatment strategies through the studied period, consisted mainly of 3-day course of Remdesivir, followed by Molnupiravir and monoclonal antibodies. In contrast to the existing data, none of them was associated with the risk for hospitalization in the multivariate analysis. This could be attributed to the fact that most of the studies mentioned above were conducted before the emergence of Omicron variant in December 2021. Moreover, randomized placebo-control studies either excluded or included only small proportion of immunocompromised patients. Finally, considering that the risk of hospitalization and death is lower with the Omicron variant, one could hypothesize that the reduction in risk obtained with these drugs may be smaller.

Our study shares strengths and limitations. It included a large population of KTRs and evaluated epidemiologic data and clinical outcomes of SARS-CoV-2 infection during the Omicron surge in Greece, excluding other periods when variants of different pathogenicity were dominant. The main limitation is that it was a single-center retrospective study, thus it does not enable us to assess long-term associations with outcomes. Moreover, there could be a risk of selection bias towards symptomatic patients, assuming that it would be less possible for asymptomatic patients to seek medical attention or even be tested for SARS-CoV-2 infection. Nevertheless, this risk is rather small considering that KTRs in our center are strongly advised to communicate with their physician in case early symptoms of the disease occur and that in Greece PCR-testing was routinely performed for several activities during that period of time.

## 5. Conclusions

In conclusion, during the third wave of the pandemic, when the Omicron variant was dominant, the clinical course of SARS-CoV-2 infection in KTRs has substantially changed, with lower rates of moderate and severe disease and a low prevalence of adverse outcomes related to COVID-19, such as SARS-CoV-2-related hospitalization and death. These observations could be explained by the reduced pathogenicity of the Omicron variant, along with the availability of preventive and therapeutic measures. Moreover, although rates of breakthrough infection with the Omicron variant remain high among vaccinated KTRs, one could hypothesize that protection against severe disease could be partially associated to vaccine-induced cellular immunity. There are no sufficient data to support the efficacy of new prophylactic and therapeutic agents in the transplanted patients. Prospective clinical trials are warranted to further elucidate the evolving pathogenesis, management, and long-term outcomes of COVID-19 in such high-risk patients as the population with organ transplants. 

## Figures and Tables

**Table 1:** Baseline characteristics of study participants.

Parameters	N = 451
Gender (male) (*n* %)	276 (61.2)
Age (years)	51.8 ± 13.7
Living Donor (*n* %)	257 (57.0)
Years from Tx	6.6 (2.7–15)
Hypertension (*n* %)	329 (72.9)
Diabetes (*n* %)	55 (12.1)
CVD (*n* %)	67 (14.8)
BMI (kg/m^2^)	25 (22.5–27.5)
Baseline creatinine (mg/dL)	1.4 (1.14–1.76)
eGFR (mL/min/1.73 m^2^)	50 (40–69)
Immunosuppression	
Tacrolimus (*n* %)	368 (81.6)
Cyclosporin A (*n* %)	49 (10.8)
MMF/MPA (*n* %)	409 (90.7)
Azathioprine (*n* %)	10 (2.2)
Everolimus (*n* %)	35 (7.8)
Steroids (*n* %)	393 (87.1)

Abbreviations: Tx, transplantation; CVD, cardiovascular disease; BMI, body mass index; eGFR, estimated glomerular filtration rate; MMF/MPA, mycophenolate mofetil/mycophenolic acid.

**Table 2:** Vaccination status and SARS-CoV-2 serology in the study population.

Parameters	N = 451
Vaccinated (*n* %)	390 (86.4)
1st dose (*n* %)	390 (86.4)
2nd dose (*n* %)	390 (86.4)
3rd dose (*n* %)	343 (76.1)
4th dose (*n* %)	148 (32.8)
Time from vaccination with	
1st dose (days)	389 (308–461)
2nd dose (days)	294 (257–382)
3rd dose (days)	188 (125–266)
4th dose (days)	27 (13–33)
Ab titer before infection (AU/mL)	33 (3.3–1205)

**Table 3:** Disease course and outcomes.

Parameters	N = 451
Radiographic findings (*n* %)	26 (6.0)
WBC (cells/10^3^)	7255 (5420–9400)
Lymphocytes (cells/10^3^)	1269 (740–1880)
Ferritin (ng/mL)	236 (120–550)
CRP (mg/L)	4 (1.4–14.5)
Reinfection (*n* %)	22 (5.0)
Hospitalization (*n* %)	51 (11.3)
COVID-19 related hospitalization (*n* %)	27 (52.9)
O_2_ treatment (*n* %)	21 (4.6)
AKI (*n* %)	18 (4.0)
ICU admission (*n* %)	8 (1.7)
Death (*n* %)	4 (0.9)

Abbreviations: WBC, white blood cells; CRP, C-reactive protein; COVID-19, corona-virus disease 2019; AKI, acute kidney injury; ICU, intensive care unit.

**Table 4:** Baseline characteristics of patients with moderate–severe disease.

Parameters	N = 26
Gender (male) (*n* %)	16 (61.5)
Age (years)	63.5 ± 9.2
Living Donor (*n* %)	25 (54.4)
Years from Tx	8.1 (3.7–13.7)
Hypertension (*n* %)	22 (84.6)
Diabetes (*n* %)	7 (26.9)
CVD (*n* %)	5 (19.2)
BMI (kg/m^2^)	26.1 (21.5–27.9)
Baseline creatinine (mg/dL)	1.3 (1.2–1.8)
eGFR (mL/min/1.73 m^2^)	46 (37–59)
Immunosuppression	
Tacrolimus (*n* %)	21 (80.8)
Cyclosporin A (*n* %)	1 (3.9)
MMF/MPA (*n* %)	22 (84.6)
Azathioprine (*n* %)	1 (3.9)
Everolimus (*n* %)	3 (11.5)
Steroids (*n* %)	24 (92.3)

Abbreviations: Tx, transplantation; CVD, cardiovascular disease; BMI, body mass index; eGFR, estimated glomerular filtration rate; MMF/MPA, mycophenolate mofetil/mycophenolic acid.

**Table 5:** Laboratory findings and outcomes of patients with moderate–severe disease.

Parameters	N = 26
WBC (cells/10^3^)	7520 (4700–10,350)
Lymphocytes (cells/10^3^)	640 (440–1020)
Ferritin (ng/mL)	652 (209–1063)
CRP (mg/L)	42 (16–69.1)
Hospitalization (*n* %)	21 (80.8)
COVID-19 related hospitalization (*n* %)	21 (100.0)
O2 treatment (*n* %)	18 (69.2)
AKI (*n* %)	3 (11.5)
ICU admission (*n* %)	5 (19.2)
Death (*n* %)	2 (7.7)

Abbreviations: WBC, white blood cells; CRP, C-reactive protein; COVID-19, corona-virus disease 2019; AKI, acute kidney injury; ICU, intensive care unit.

**Table 6:** Therapeutic management of COVID-19 during the Omicron surge.

Parameters	N = 451
Immunosuppression handling	
MMF/MPA discontinuation (*n* = 409) (*n* %)	338 (82.6)
MMF/MPA dose reduction (*n* %)	71 (17.4)
Tacrolimus trough levels < 6 ng/mL (*n* = 386) (*n* %)	150 (40.8)
Dexamethasone (*n* %)	20 (4.4)
Remdesivir (3-day) (*n* %)	183 (40.6)
Remdesivir (5-day) (*n* %)	18 (3.9)
Tocilizumab (*n* %)	2 (0.4)
Monoclonal Abs (*n* %)	47 (10.4)
Molnupiravir (*n* %)	97 (21.5)
Antibiotics (*n* %)	33 (7.3)
Anticoagulants (*n* %)	391 (86.6)

Abbreviations: MMF/MPA, mycophenolate mofetil/mycophenolic acid.

## Data Availability

Data supporting reported results are available upon request from the corresponding author.

## References

[1] Akalin E., Azzi Y., Bartash R., Seethamraju H., Parides M., Hemmige V., Ross M., Forest S., Goldstein Y.D., Ajaimy M. (2020). COVID-19 and Kidney Transplantation. N. Engl. J. Med..

[2] Ao G., Wang Y., Qi X., Nasr B., Bao M., Gao M., Sun Y., Xie D. (2021). The association between severe or death COVID-19 and solid organ transplantation: A systematic review and meta-analysis. Transplant. Rev..

[3] Azzi Y., Bartash R., Scalea J., Loarte-Campos P., Akalin E. (2021). COVID-19 and Solid Organ Transplantation: A Review Article. Transplantation.

[4] Danziger-Isakov L., Blumberg E.A., Manuel O., Sester M. (2021). Impact of COVID-19 in solid organ transplant recipients. Am. J. Transplant..

[5] Tschopp J., L’Huillier A.G., Mombelli M., Mueller N.J., Khanna N., Garzoni C., Meloni D., Papadimitriou-Olivgeris M., Neofytos D., Hirsch H.H. (2020). First experience of SARS-CoV-2 infections in solid organ transplant recipients in the Swiss Transplant Cohort Study. Am. J. Transplant..

[6] Andrews N., Stowe J., Kirsebom F., Toffa S., Rickeard T., Gallagher E., Gower C., Kall M., Groves N., O’Connell A.-M. (2022). COVID-19 Vaccine Effectiveness against the Omicron (B.1.1.529) Variant. N. Engl. J. Med..

[7] Cochran W., Shah P., Barker L., Langlee J., Freed K., Boyer L., Anderson R.S., Belden M., Bannon J., Kates O.S. (2022). COVID-19 Clinical Outcomes in Solid Organ Transplant Recipients During the Omicron Surge. Transplantation.

[8] Villanego F., Vigara L.A., Alonso M., Orellana C., Gómez A.M., Eady M., Sánchez M.G., Gómez R., García T., Mazuecos A. (2022). Trends in COVID-19 Outcomes in Kidney Transplant Recipients during the Period of Omicron Variant Predominance. Transplantation.

[9] Korth J., Jahn M., Dorsch O., Anastasiou O.E., Sorge-Hädicke B., Eisenberger U., Gäckler A., Dittmer U., Witzke O., Wilde B. (2021). Impaired Humoral Response in Renal Transplant Recipients to SARS-CoV-2 Vaccination with BNT162b2 (Pfizer-BioNTech). Viruses.

[10] Grupper A., Rabinowich L., Schwartz D., Schwartz I.F., Ben-Yehoyada M., Shashar M., Katchman E., Halperin T., Turner D., Goykhman Y. (2021). Reduced humoral response to mRNA SARS-CoV-2 BNT162b2 vaccine in kidney transplant recipients without prior exposure to the virus. Am. J. Transplant..

[11] Kamar N., Abravanel F., Marion O., Couat C., Izopet J., Del Bello A. (2021). Three Doses of an mRNA Covid-19 Vaccine in Solid-Organ Transplant Recipients. N. Engl. J. Med..

[12] Aslam S., Adler E., Mekeel K., Little S.J. (2021). Clinical effectiveness of COVID-19 vaccination in solid organ transplant recipients. Transpl. Infect. Dis..

[13] Aslam S., Liu J., Sigler R., Syed R.R., Tu X.M., Little S.J., De Gruttola V. (2022). Coronavirus disease 2019 vaccination is protective of clinical disease in solid organ transplant recipients. Transpl. Infect. Dis..

[14] Perkmann T., Perkmann-Nagele N., Breyer M.K., Breyer-Kohansal R., Burghuber O.C., Hartl S., Aletaha D., Sieghart D., Quehenberger P., Marculescu R. (2020). Side-by-Side Comparison of Three Fully Automated SARS-CoV-2 Antibody Assays with a Focus on Specificity. Clin. Chem..

[15] Maggiore U., Abramowicz D., Crespo M., Mariat C., Mjoen G., Peruzzi L., Sever M.S., Oniscu G.C., Hilbrands L., Watschinger B. (2020). How should I manage immunosuppression in a kidney transplant patient with COVID-19? An ERA-EDTA DESCARTES expert opinion. Nephrol. Dial. Transplant..

[16] López V., Vázquez T., Alonso-Titos J., Cabello M., Alonso A., Beneyto I., Crespo M., Díaz-Corte C., Franco A., González-Roncero F. (2020). Recommendations on management of the SARS-CoV-2 coronavirus pandemic (COVID-19) in kidney transplant patients. Nefrologia.

[17] Menni C., Valdes A.M., Polidori L., Antonelli M., Penamakuri S., Nogal A., Louca P., May A., Figueiredo J.C., Hu C. (2022). Symptom prevalence, duration, and risk of hospital admission in individuals infected with SARS-CoV-2 during periods of omicron and delta variant dominance: A prospective observational study from the ZOE COVID Study. Lancet.

[18] Bertrand D., Laurent C., Lemée V., Lebourg L., Hanoy M., Le Roy F., Nezam D., Pruteanu D., Grange S., de Nattes T. (2022). Efficacy of anti-SARS-CoV-2 monoclonal antibody prophylaxis and vaccination on the Omicron variant of COVID-19 in kidney transplant recipients. Kidney Int..

[19] Garcia-Beltran W.F., St Denis K.J., Hoelzemer A., Lam E.C., Nitido A.D., Sheehan M.L., Berrios C., Ofoman O., Chang C.C., Hauser B.M. (2022). mRNA-based COVID-19 vaccine boosters induce neutralizing immunity against SARS-CoV-2 Omicron variant. Cell.

[20] Al Jurdi A., Gassen R.B., Borges T.J., Lape I.T., Morena L., Efe O., Solhjou Z., El Fekih R., Deban C., Bohan B. (2022). Suboptimal antibody response against SARS-CoV-2 Omicron variant after third dose of mRNA vaccine in kidney transplant recipients. Kidney Int..

[21] Kumar D., Hu Q., Samson R., Ferreira V.H., Hall V.G., Ierullo M., Majchrzak-Kita B., Hardy W., Gingras A.-C., Humar A. (2022). Neutralization against Omicron variant in transplant recipients after three doses of mRNA vaccine. Am. J. Transplant..

[22] Tarke A., Coelho C.H., Zhang Z., Dan J.M., Yu E.D., Methot N., Bloom N.I., Goodwin B., Phillips E., Mallal S. (2022). SARS-CoV-2 vaccination induces immunological T cell memory able to cross-recognize variants from Alpha to Omicron. Cell.

[23] Sarrell B.A., Bloch K., El Chediak A., Kumm K., Tracy K., Forbes R.C., Langone A., Thomas L., Schlendorf K., Trindade A.J. (2022). Monoclonal antibody treatment for COVID-19 in solid organ transplant recipients. Transpl. Infect. Dis..

[24] Fernandes G., Devresse A., Scohy A., Yombi J.C., Belkhir L., De Greef J., Darius T., Buemi A., Kabamba B., Goffin E. (2022). Monoclonal Antibody Therapy for SARS-CoV-2 Infection in Kidney Transplant Recipients: A Case Series from Belgium. Transplantation.

[25] Pinchera B., Buonomo A.R., Scotto R., Carrano R., Salemi F., Galluccio F., Guarino M., Viceconte G., Moriello N.S., Giaccone A. (2022). Sotrovimab in Solid Organ Transplant Patients with Early, Mild/Moderate SARS-CoV-2 Infection: A Single-center Experience. Transplantation.

[26] Klein E.J., Hardesty A., Vieira K., Farmakiotis D. (2022). Use of anti-spike monoclonal antibodies in kidney transplant recipients with COVID-19: Efficacy, ethnic and racial disparities. Am. J. Transplant..

[27] Papadimitriou-Olivgeris M., Cipriano A., Guggisberg N., Kroemer M., Tschopp J., Manuel O., Golshayan D. (2022). Outcome of COVID-19 in Kidney Transplant Recipients Through the SARS-CoV-2 Variants Eras: Role of Anti-SARS-CoV-2 Monoclonal Antibodies. Transpl. Int..

[28] Gottlieb R.L., Vaca C.E., Paredes R., Mera J., Webb B.J., Perez G., Oguchi G., Ryan P., Nielsen B.U., Brown M. (2022). Early Remdesivir to Prevent Progression to Severe COVID-19 in Outpatients. N. Engl. J. Med..

[29] Jayk Bernal A., Gomes da Silva M.M., Musungaie D.B., Kovalchuk E., Gonzalez A., Delos Reyes V., Martín-Quirós A., Caraco Y., Williams-Diaz A., Brown M.L. (2022). Molnupiravir for Oral Treatment of COVID-19 in Nonhospitalized Patients. N. Engl. J. Med..

